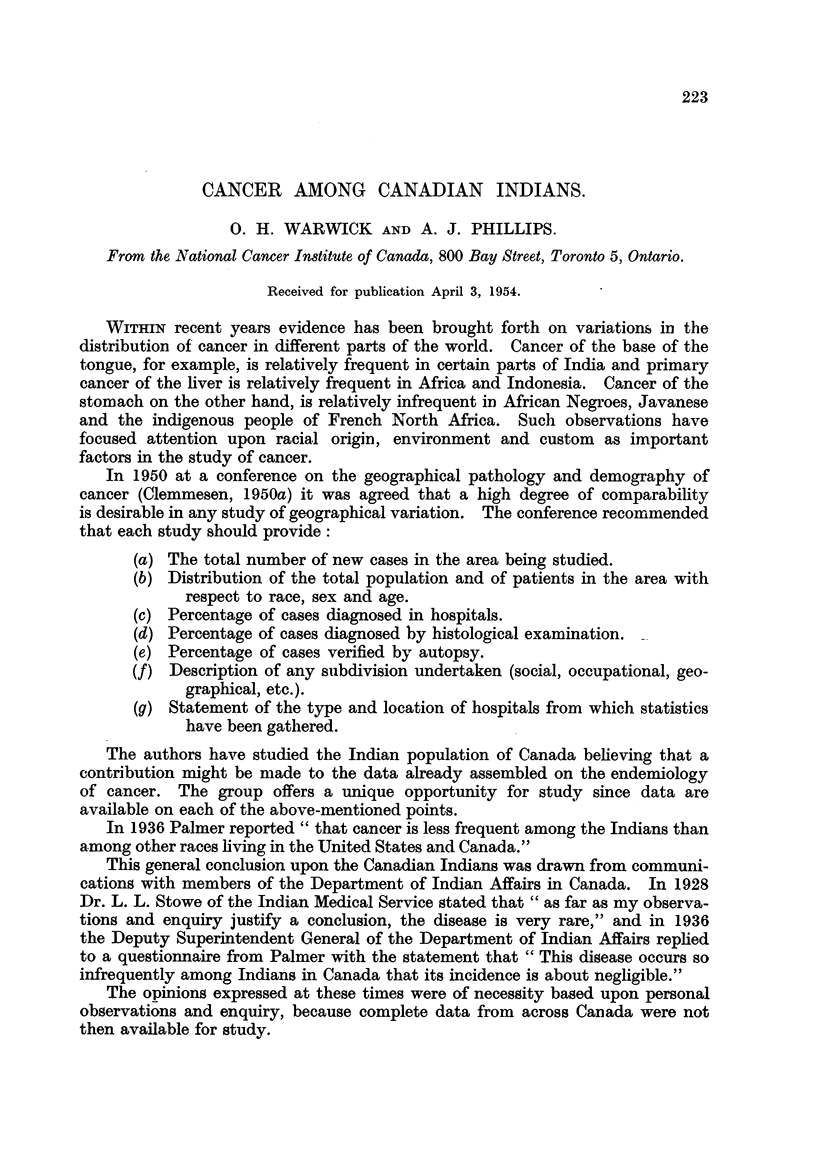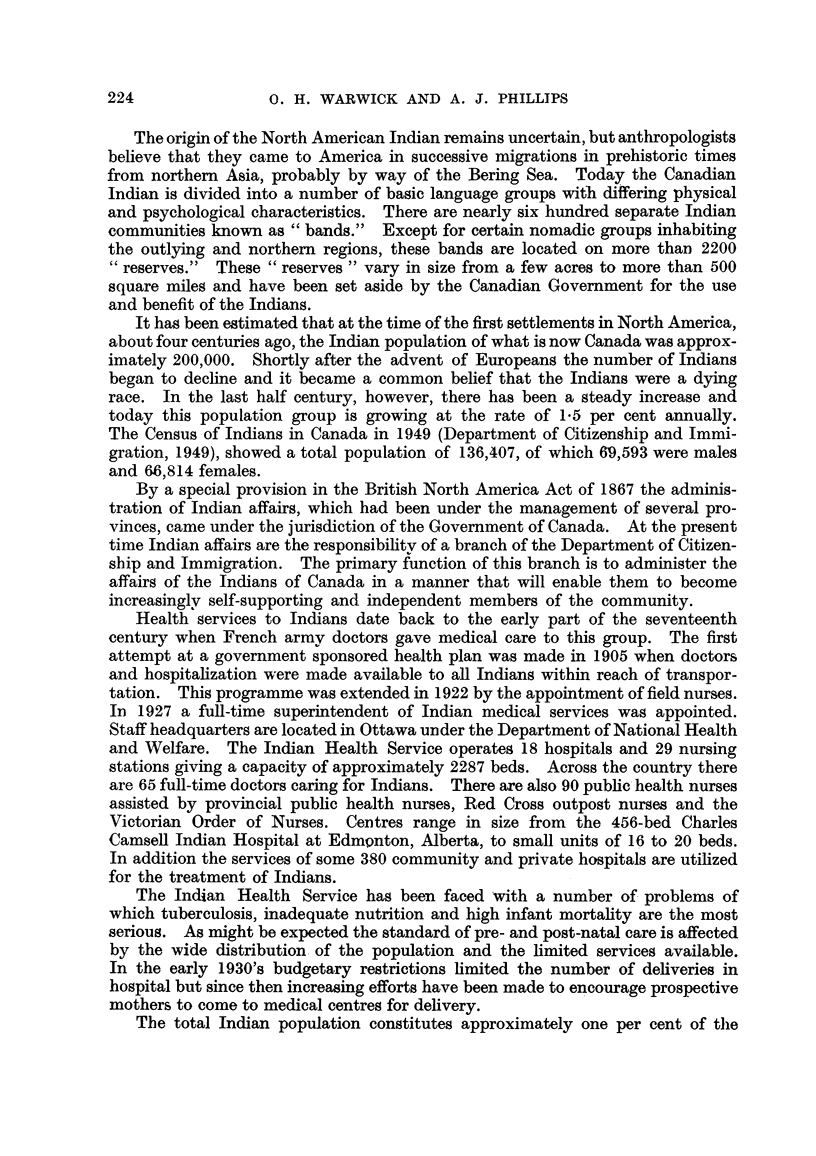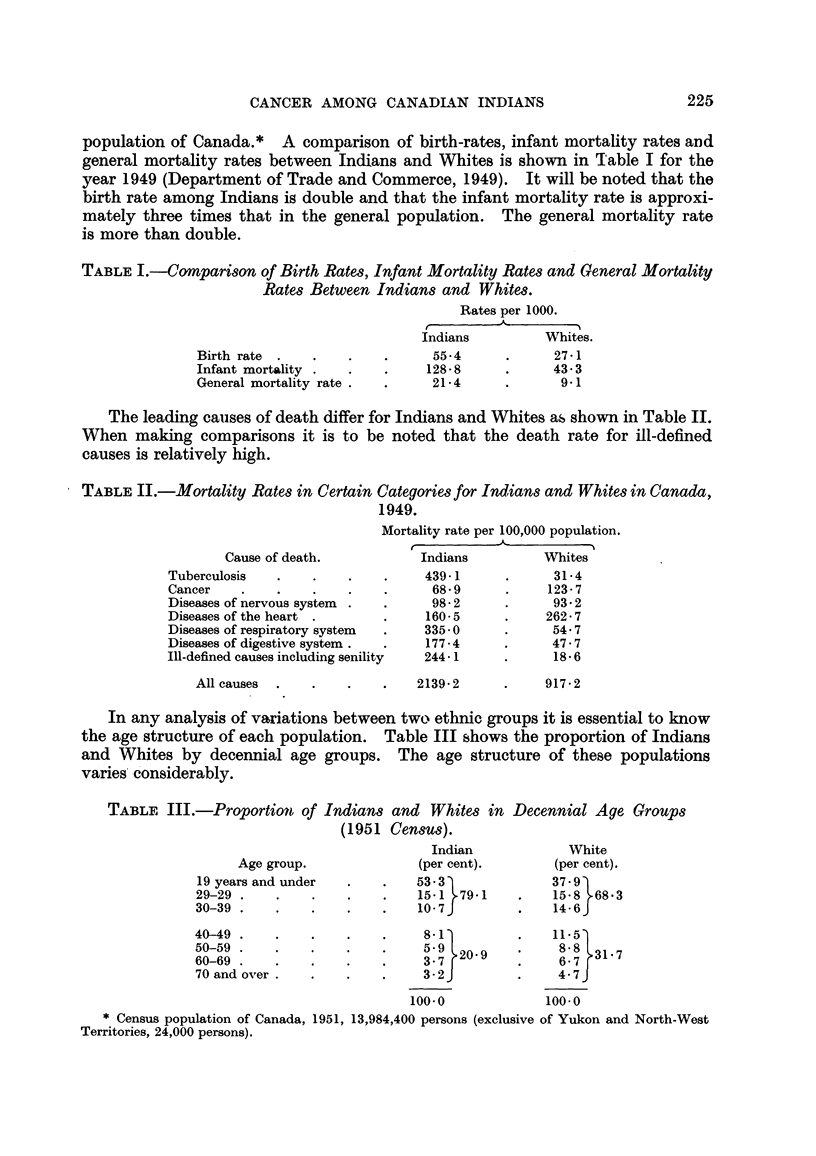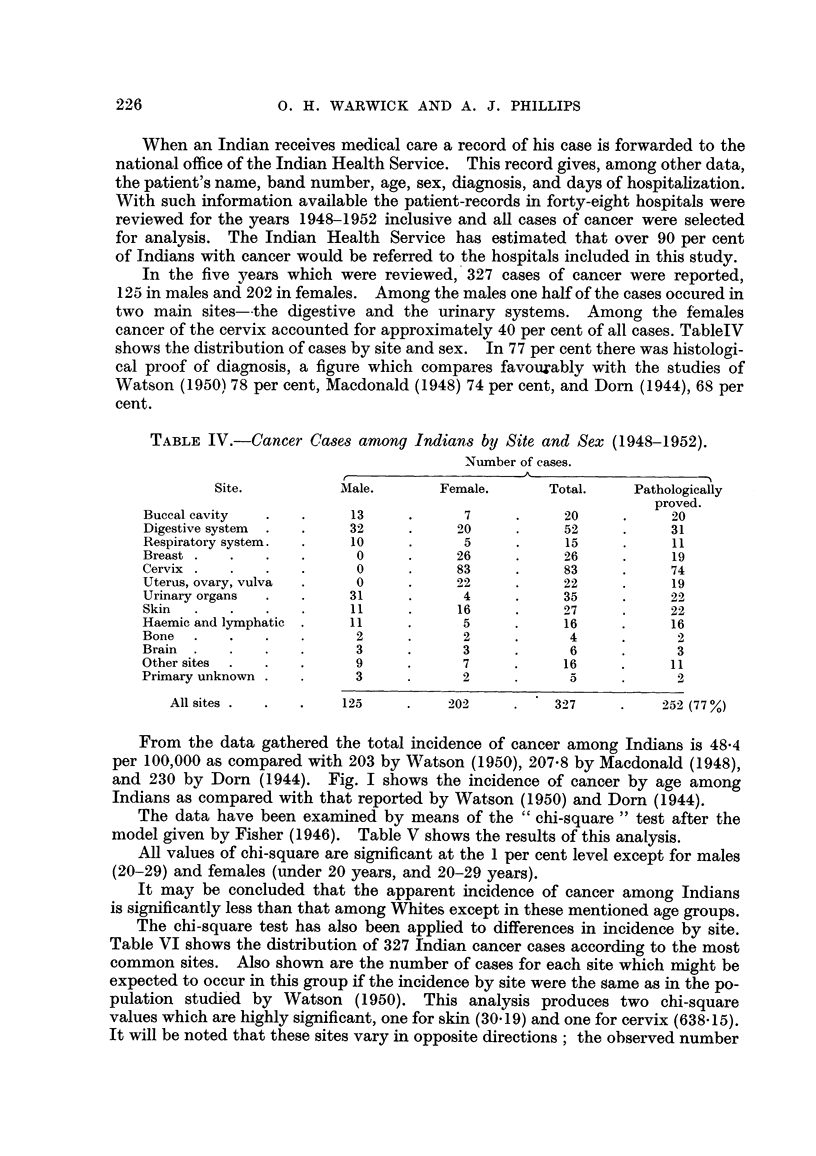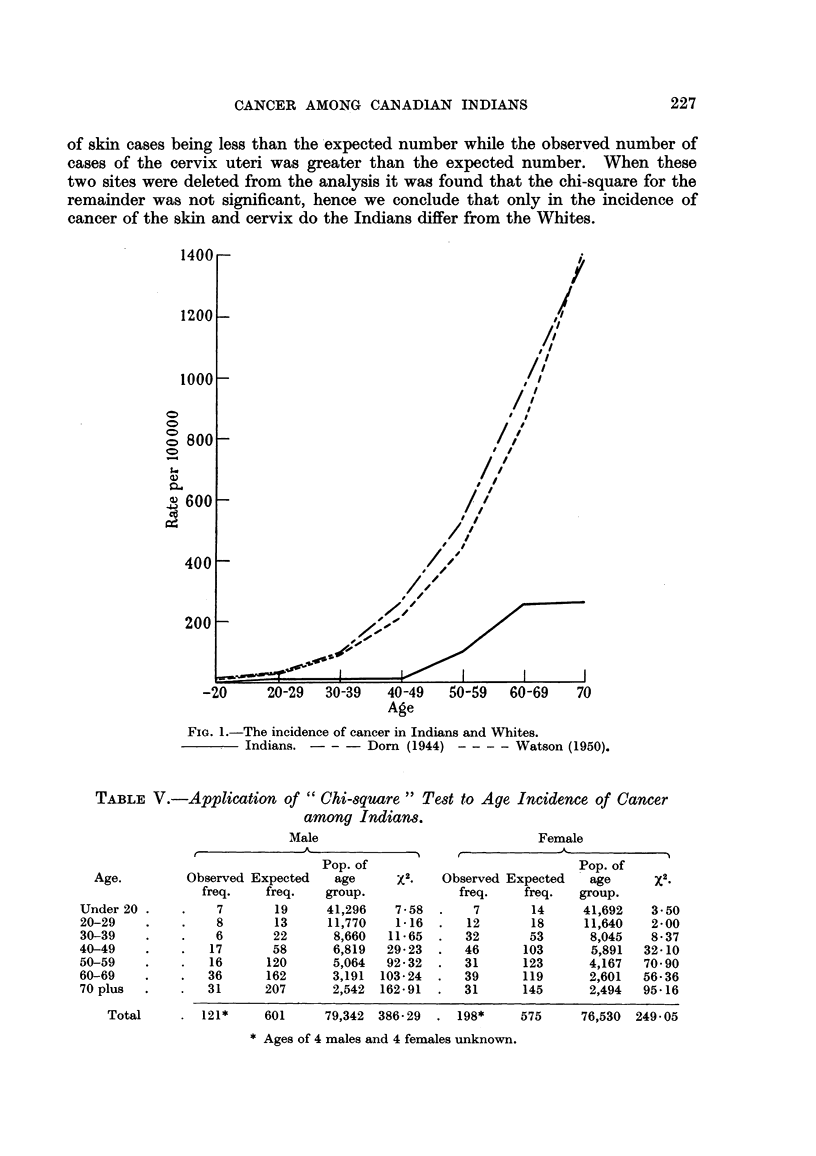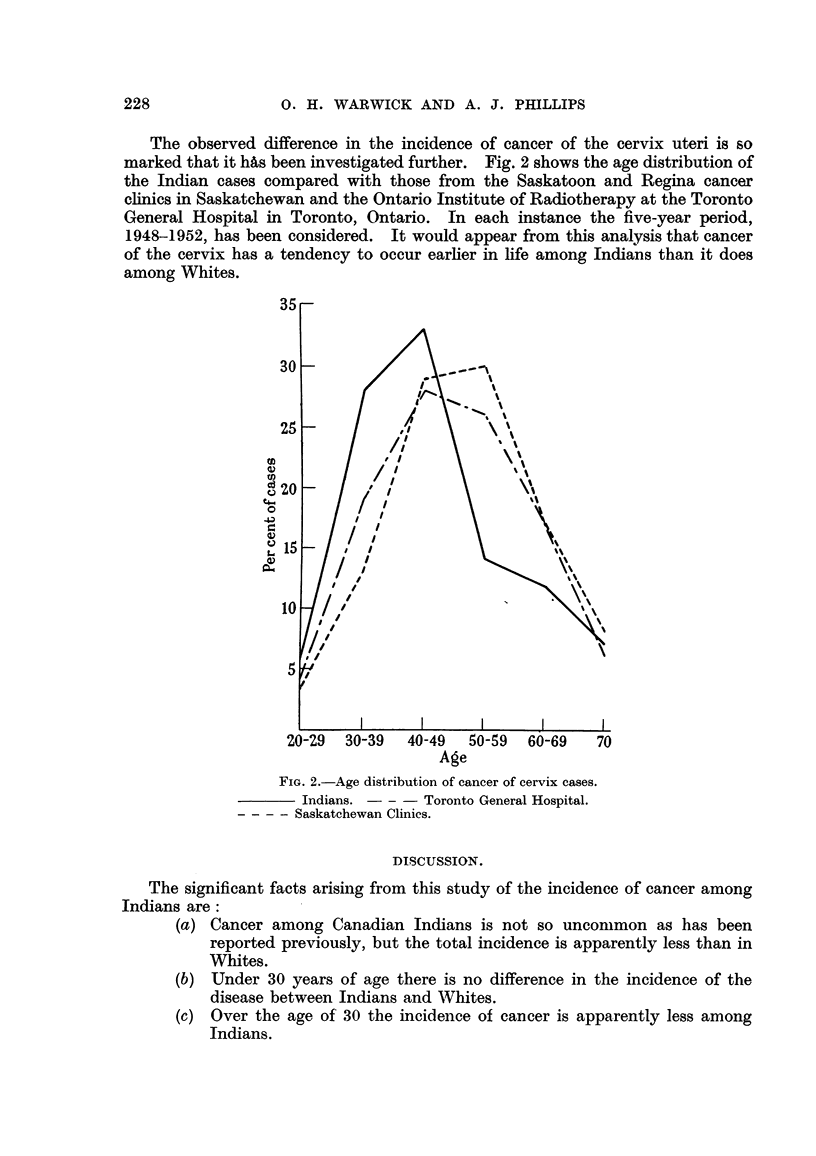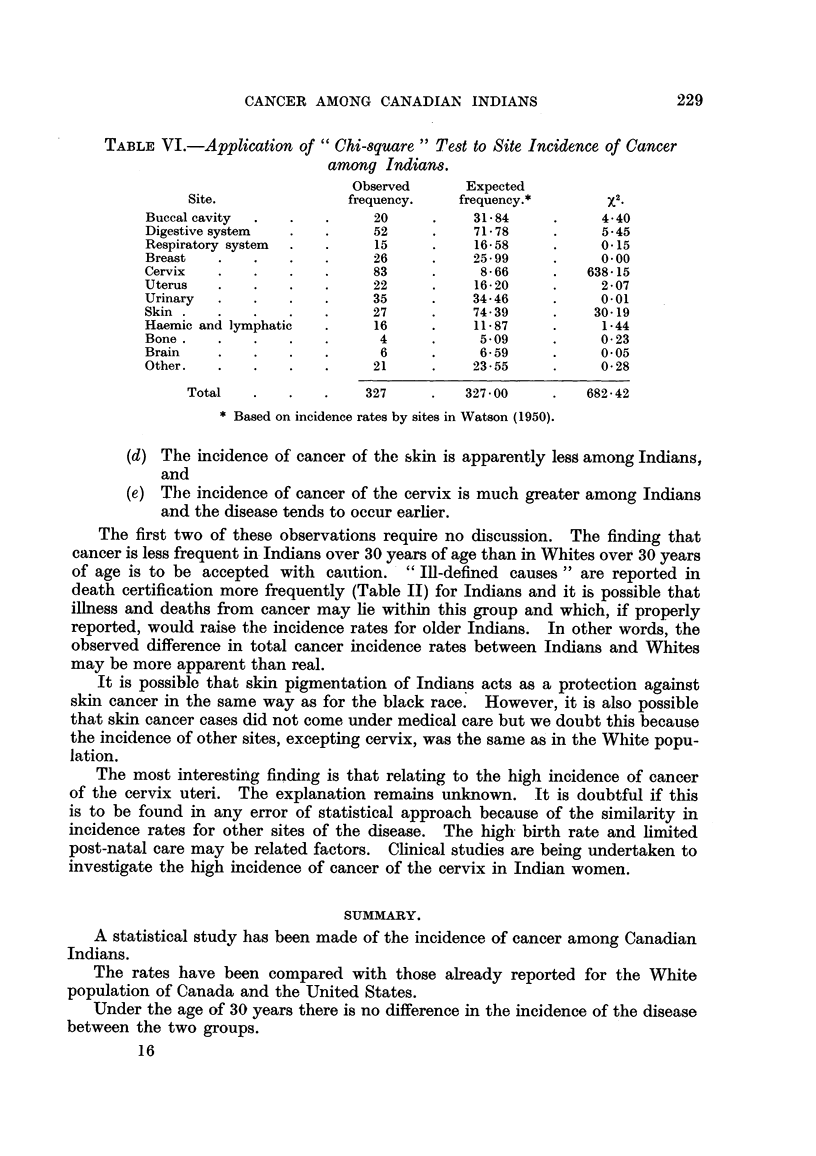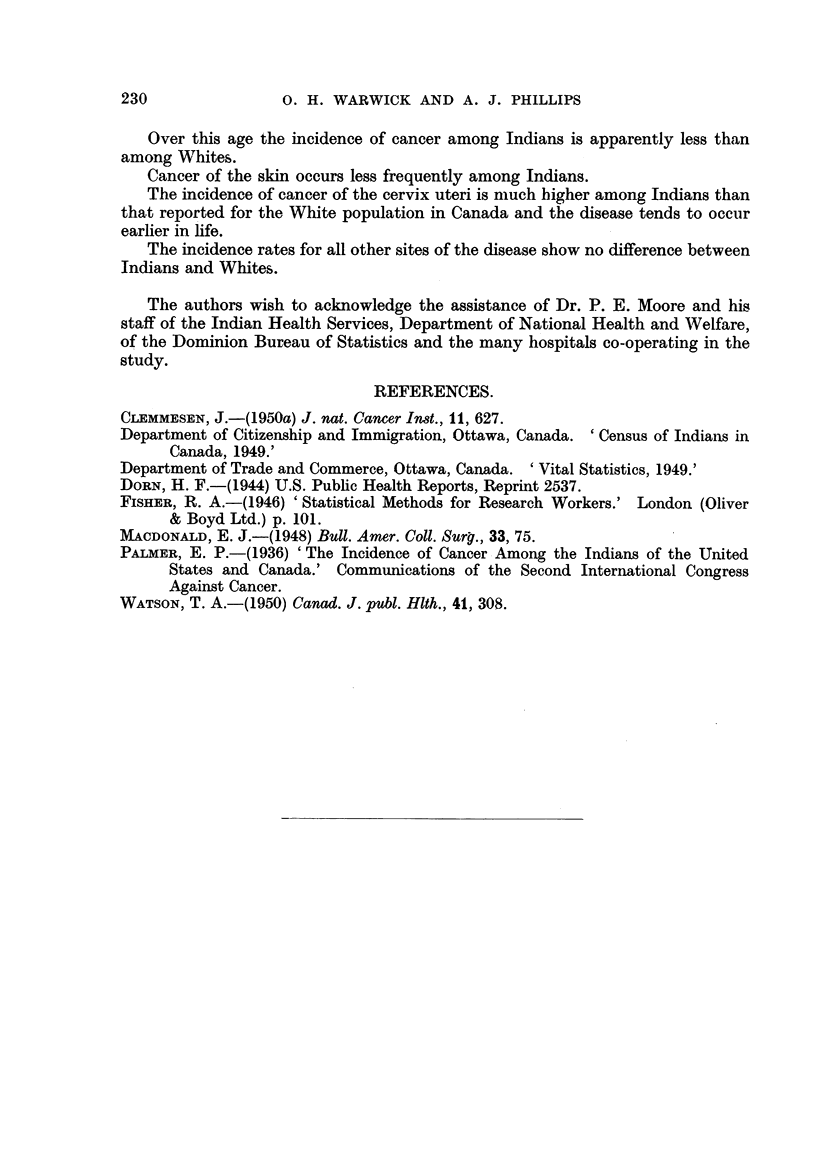# Cancer Among Canadian Indians

**DOI:** 10.1038/bjc.1954.22

**Published:** 1954-06

**Authors:** O. H. Warwick, A. J. Phillips


### CANCER AMONG CANADIAN INDIANS.

O. H. WARWICK AND A. J. PHILLIPS.

*From the National Cancer Institute of Canada, 800 Bay Street, Toronto 5, Ontario.*

Received for publication April 3, 1954.

WITHIN recent years evidence has been brought forth on variations in the distribution of cancer in different parts of the world. Cancer of the base of the tongue, for example, is relatively frequent in certain parts of India and primary cancer of the liver is relatively frequent in Africa and Indonesia. Cancer of the stomach on the other hand, is relatively infrequent in African Negroes, Javanese and the indigenous people of French North Africa. Such observations have focused attention upon racial origin, environment and custom as important factors in the study of cancer.

In 1950 at a conference on the geographical pathology and demography of cancer (Clemmesen, 1950*a*) it was agreed that a high degree of comparability is desirable in any study of geographical variation. The conference recommended that each study should provide :

(*a*) The total number of new cases in the area being studied.
(*b*) Distribution of the total population and of patients in the area with respect to race, sex and age.
(*c*) Percentage of cases diagnosed in hospitals.
(*d*) Percentage of cases diagnosed by histological examination.
(*e*) Percentage of cases verified by autopsy.
(*f*) Description of any subdivision undertaken (social, occupational, geographical, etc.).
(*g*) Statement of the type and location of hospitals from which statistics have been gathered.

The authors have studied the Indian population of Canada believing that a contribution might be made to the data already assembled on the endemiology of cancer. The group offers a unique opportunity for study since data are available on each of the above-mentioned points.

In 1936 Palmer reported " that cancer is less frequent among the Indians than among other races living in the United States and Canada."

This general conclusion upon the Canadian Indians was drawn from communications with members of the Department of Indian Affairs in Canada. In 1928 Dr. L. L. Stowe of the Indian Medical Service stated that " as far as my observations and enquiry justify a conclusion, the disease is very rare," and in 1936 the Deputy Superintendent General of the Department of Indian Affairs replied to a questionnaire from Palmer with the statement that " This disease occurs so infrequently among Indians in Canada that its incidence is about negligible."

The opinions expressed at these times were of necessity based upon personal observations and enquiry, because complete data from across Canada were not then available for study.

The origin of the North American Indian remains uncertain, but anthropologists believe that they came to America in successive migrations in prehistoric times from northern Asia, probably by way of the Bering Sea. Today the Canadian Indian is divided into a number of basic language groups with differing physical and psychological characteristics. There are nearly six hundred separate Indian communities known as " bands." Except for certain nomadic groups inhabiting the outlying and northern regions, these bands are located on more than 2200 " reserves." These " reserves " vary in size from a few acres to more than 500 square miles and have been set aside by the Canadian Government for the use and benefit of the Indians.

It has been estimated that at the time of the first settlements in North America, about four centuries ago, the Indian population of what is now Canada was approximately 200,000. Shortly after the advent of Europeans the number of Indians began to decline and it became a common belief that the Indians were a dying race. In the last half century, however, there has been a steady increase and today this population group is growing at the rate of 1·5 per cent annually. The Census of Indians in Canada in 1949 (Department of Citizenship and Immigration, 1949), showed a total population of 136,407, of which 69,593 were males and 66,814 females.

By a special provision in the British North America Act of 1867 the administration of Indian affairs, which had been under the management of several provinces, came under the jurisdiction of the Government of Canada. At the present time Indian affairs are the responsibility of a branch of the Department of Citizenship and Immigration. The primary function of this branch is to administer the affairs of the Indians of Canada in a manner that will enable them to become increasingly self-supporting and independent members of the community.

Health services to Indians date back to the early part of the seventeenth century when French army doctors gave medical care to this group. The first attempt at a government sponsored health plan was made in 1905 when doctors and hospitalization were made available to all Indians within reach of transportation. This programme was extended in 1922 by the appointment of field nurses. In 1927 a full-time superintendent of Indian medical services was appointed. Staff headquarters are located in Ottawa under the Department of National Health and Welfare. The Indian Health Service operates 18 hospitals and 29 nursing stations giving a capacity of approximately 2287 beds. Across the country there are 65 full-time doctors caring for Indians. There are also 90 public health nurses assisted by provincial public health nurses, Red Cross outpost nurses and the Victorian Order of Nurses. Centres range in size from the 456-bed Charles Camsell Indian Hospital at Edmonton, Alberta, to small units of 16 to 20 beds. In addition the services of some 380 community and private hospitals are utilized for the treatment of Indians.

The Indian Health Service has been faced with a number of problems of which tuberculosis, inadequate nutrition and high infant mortality are the most serious. As might be expected the standard of pre- and post-natal care is affected by the wide distribution of the population and the limited services available. In the early 1930's budgetary restrictions limited the number of deliveries in hospital but since then increasing efforts have been made to encourage prospective mothers to come to medical centres for delivery.

The total Indian population constitutes approximately one per cent of the population of Canada.* A comparison of birth-rates, infant mortality rates and general mortality rates between Indians and Whites is shown in Table I for the year 1949 (Department of Trade and Commerce, 1949). It will be noted that the birth rate among Indians is double and that the infant mortality rate is approximately three times that in the general population. The general mortality rate is more than double.

TABLE I.—*Comparison of Birth Rates, Infant Mortality Rates and General Mortality Rates Between Indians and Whites.*

| | Rates per 1000. Indians | Whites. |
|---|---|---|
| Birth rate | 55·4 | 27·1 |
| Infant mortality | 128·8 | 43·3 |
| General mortality rate | 21·4 | 9·1 |

The leading causes of death differ for Indians and Whites as shown in Table II. When making comparisons it is to be noted that the death rate for ill-defined causes is relatively high.

TABLE II.—*Mortality Rates in Certain Categories for Indians and Whites in Canada,* 1949.

| Cause of death. | Mortality rate per 100,000 population. Indians | Whites |
|---|---|---|
| Tuberculosis | 439·1 | 31·4 |
| Cancer | 68·9 | 123·7 |
| Diseases of nervous system | 98·2 | 93·2 |
| Diseases of the heart | 160·5 | 262·7 |
| Diseases of respiratory system | 335·0 | 54·7 |
| Diseases of digestive system | 177·4 | 47·7 |
| Ill-defined causes including senility | 244·1 | 18·6 |
| All causes | 2139·2 | 917·2 |

In any analysis of variations between two ethnic groups it is essential to know the age structure of each population. Table III shows the proportion of Indians and Whites by decennial age groups. The age structure of these populations varies considerably.

TABLE III.—*Proportion of Indians and Whites in Decennial Age Groups (1951 Census).*

| Age group. | Indian (per cent). | | White (per cent). | |
|---|---|---|---|---|
| 19 years and under | 53·3 | 79·1 | 37·9 | 68·3 |
| 29–29 | 15·1 | | 15·8 | |
| 30–39 | 10·7 | | 14·6 | |
| 40–49 | 8·1 | 20·9 | 11·5 | 31·7 |
| 50–59 | 5·9 | | 8·8 | |
| 60–69 | 3·7 | | 6·7 | |
| 70 and over | 3·2 | | 4·7 | |
| | 100·0 | | 100·0 | |

* Census population of Canada, 1951, 13,984,400 persons (exclusive of Yukon and North-West Territories, 24,000 persons).

When an Indian receives medical care a record of his case is forwarded to the national office of the Indian Health Service. This record gives, among other data, the patient's name, band number, age, sex, diagnosis, and days of hospitalization. With such information available the patient-records in forty-eight hospitals were reviewed for the years 1948–1952 inclusive and all cases of cancer were selected for analysis. The Indian Health Service has estimated that over 90 per cent of Indians with cancer would be referred to the hospitals included in this study.

In the five years which were reviewed, 327 cases of cancer were reported, 125 in males and 202 in females. Among the males one half of the cases occured in two main sites—the digestive and the urinary systems. Among the females cancer of the cervix accounted for approximately 40 per cent of all cases. TableIV shows the distribution of cases by site and sex. In 77 per cent there was histological proof of diagnosis, a figure which compares favourably with the studies of Watson (1950) 78 per cent, Macdonald (1948) 74 per cent, and Dorn (1944), 68 per cent.

TABLE IV.—*Cancer Cases among Indians by Site and Sex* (1948–1952).

| Site. | Number of cases. Male. | Female. | Total. | Pathologically proved. |
|---|---|---|---|---|
| Buccal cavity | 13 | 7 | 20 | 20 |
| Digestive system | 32 | 20 | 52 | 31 |
| Respiratory system | 10 | 5 | 15 | 11 |
| Breast | 0 | 26 | 26 | 19 |
| Cervix | 0 | 83 | 83 | 74 |
| Uterus, ovary, vulva | 0 | 22 | 22 | 19 |
| Urinary organs | 31 | 4 | 35 | 22 |
| Skin | 11 | 16 | 27 | 22 |
| Haemic and lymphatic | 11 | 5 | 16 | 16 |
| Bone | 2 | 2 | 4 | 2 |
| Brain | 3 | 3 | 6 | 3 |
| Other sites | 9 | 7 | 16 | 11 |
| Primary unknown | 3 | 2 | 5 | 2 |
| All sites | 125 | 202 | 327 | 252 (77%) |

From the data gathered the total incidence of cancer among Indians is 48·4 per 100,000 as compared with 203 by Watson (1950), 207·8 by Macdonald (1948), and 230 by Dorn (1944). Fig. I shows the incidence of cancer by age among Indians as compared with that reported by Watson (1950) and Dorn (1944).

The data have been examined by means of the " chi-square " test after the model given by Fisher (1946). Table V shows the results of this analysis.

All values of chi-square are significant at the 1 per cent level except for males (20–29) and females (under 20 years, and 20–29 years).

It may be concluded that the apparent incidence of cancer among Indians is significantly less than that among Whites except in these mentioned age groups.

The chi-square test has also been applied to differences in incidence by site. Table VI shows the distribution of 327 Indian cancer cases according to the most common sites. Also shown are the number of cases for each site which might be expected to occur in this group if the incidence by site were the same as in the population studied by Watson (1950). This analysis produces two chi-square values which are highly significant, one for skin (30·19) and one for cervix (638·15). It will be noted that these sites vary in opposite directions ; the observed number of skin cases being less than the expected number while the observed number of cases of the cervix uteri was greater than the expected number. When these two sites were deleted from the analysis it was found that the chi-square for the remainder was not significant, hence we conclude that only in the incidence of cancer of the skin and cervix do the Indians differ from the Whites.

FIG. 1.—The incidence of cancer in Indians and Whites.
——— Indians. — — — Dorn (1944) - - - - Watson (1950).

TABLE V.—*Application of " Chi-square " Test to Age Incidence of Cancer among Indians.*

| Age. | Male: Observed freq. | Male: Expected freq. | Male: Pop. of age group. | Male: $\chi^2$. | Female: Observed freq. | Female: Expected freq. | Female: Pop. of age group. | Female: $\chi^2$. |
|---|---|---|---|---|---|---|---|---|
| Under 20 | 7 | 19 | 41,296 | 7·58 | 7 | 14 | 41,692 | 3·50 |
| 20–29 | 8 | 13 | 11,770 | 1·16 | 12 | 18 | 11,640 | 2·00 |
| 30–39 | 6 | 22 | 8,660 | 11·65 | 32 | 53 | 8,045 | 8·37 |
| 40–49 | 17 | 58 | 6,819 | 29·23 | 46 | 103 | 5,891 | 32·10 |
| 50–59 | 16 | 120 | 5,064 | 92·32 | 31 | 123 | 4,167 | 70·90 |
| 60–69 | 36 | 162 | 3,191 | 103·24 | 39 | 119 | 2,601 | 56·36 |
| 70 plus | 31 | 207 | 2,542 | 162·91 | 31 | 145 | 2,494 | 95·16 |
| Total | 121* | 601 | 79,342 | 386·29 | 198* | 575 | 76,530 | 249·05 |

* Ages of 4 males and 4 females unknown.

The observed difference in the incidence of cancer of the cervix uteri is so marked that it hås been investigated further. Fig. 2 shows the age distribution of the Indian cases compared with those from the Saskatoon and Regina cancer clinics in Saskatchewan and the Ontario Institute of Radiotherapy at the Toronto General Hospital in Toronto, Ontario. In each instance the five-year period, 1948–1952, has been considered. It would appear from this analysis that cancer of the cervix has a tendency to occur earlier in life among Indians than it does among Whites.

FIG. 2.—Age distribution of cancer of cervix cases.
——— Indians. — — — Toronto General Hospital.
- - - - Saskatchewan Clinics.

DISCUSSION.

The significant facts arising from this study of the incidence of cancer among Indians are :

(*a*) Cancer among Canadian Indians is not so uncommon as has been reported previously, but the total incidence is apparently less than in Whites.

(*b*) Under 30 years of age there is no difference in the incidence of the disease between Indians and Whites.

(*c*) Over the age of 30 the incidence of cancer is apparently less among Indians.

TABLE VI.—*Application of " Chi-square " Test to Site Incidence of Cancer among Indians.*

| Site. | Observed frequency. | Expected frequency.* | $\chi^2$. |
|---|---|---|---|
| Buccal cavity | 20 | 31·84 | 4·40 |
| Digestive system | 52 | 71·78 | 5·45 |
| Respiratory system | 15 | 16·58 | 0·15 |
| Breast | 26 | 25·99 | 0·00 |
| Cervix | 83 | 8·66 | 638·15 |
| Uterus | 22 | 16·20 | 2·07 |
| Urinary | 35 | 34·46 | 0·01 |
| Skin | 27 | 74·39 | 30·19 |
| Haemic and lymphatic | 16 | 11·87 | 1·44 |
| Bone | 4 | 5·09 | 0·23 |
| Brain | 6 | 6·59 | 0·05 |
| Other | 21 | 23·55 | 0·28 |
| Total | 327 | 327·00 | 682·42 |

* Based on incidence rates by sites in Watson (1950).

(*d*) The incidence of cancer of the skin is apparently less among Indians, and

(*e*) The incidence of cancer of the cervix is much greater among Indians and the disease tends to occur earlier.

The first two of these observations require no discussion. The finding that cancer is less frequent in Indians over 30 years of age than in Whites over 30 years of age is to be accepted with caution. " Ill-defined causes " are reported in death certification more frequently (Table II) for Indians and it is possible that illness and deaths from cancer may lie within this group and which, if properly reported, would raise the incidence rates for older Indians. In other words, the observed difference in total cancer incidence rates between Indians and Whites may be more apparent than real.

It is possible that skin pigmentation of Indians acts as a protection against skin cancer in the same way as for the black race. However, it is also possible that skin cancer cases did not come under medical care but we doubt this because the incidence of other sites, excepting cervix, was the same as in the White population.

The most interesting finding is that relating to the high incidence of cancer of the cervix uteri. The explanation remains unknown. It is doubtful if this is to be found in any error of statistical approach because of the similarity in incidence rates for other sites of the disease. The high birth rate and limited post-natal care may be related factors. Clinical studies are being undertaken to investigate the high incidence of cancer of the cervix in Indian women.

### SUMMARY.

A statistical study has been made of the incidence of cancer among Canadian Indians.

The rates have been compared with those already reported for the White population of Canada and the United States.

Under the age of 30 years there is no difference in the incidence of the disease between the two groups.

Over this age the incidence of cancer among Indians is apparently less than among Whites.

Cancer of the skin occurs less frequently among Indians.

The incidence of cancer of the cervix uteri is much higher among Indians than that reported for the White population in Canada and the disease tends to occur earlier in life.

The incidence rates for all other sites of the disease show no difference between Indians and Whites.

The authors wish to acknowledge the assistance of Dr. P. E. Moore and his staff of the Indian Health Services, Department of National Health and Welfare, of the Dominion Bureau of Statistics and the many hospitals co-operating in the study.

### REFERENCES.

CLEMMESEN, J.—(1950*a*) *J. nat. Cancer Inst.*, **11**, 627.

Department of Citizenship and Immigration, Ottawa, Canada. 'Census of Indians in Canada, 1949.'

Department of Trade and Commerce, Ottawa, Canada. 'Vital Statistics, 1949.'

DORN, H. F.—(1944) U.S. Public Health Reports, Reprint 2537.

FISHER, R. A.—(1946) 'Statistical Methods for Research Workers.' London (Oliver & Boyd Ltd.) p. 101.

MACDONALD, E. J.—(1948) *Bull. Amer. Coll. Surg.*, **33**, 75.

PALMER, E. P.—(1936) 'The Incidence of Cancer Among the Indians of the United States and Canada.' Communications of the Second International Congress Against Cancer.

WATSON, T. A.—(1950) *Canad. J. publ. Hlth.*, **41**, 308.